# Revolution in Gene Medicine Therapy and Genome Surgery

**DOI:** 10.3390/genes9120575

**Published:** 2018-11-26

**Authors:** David J. Jiang, Christine L. Xu, Stephen H. Tsang

**Affiliations:** 1 Jonas Children’s Vision Care and Bernard & Shirlee Brown Glaucoma Laboratory, Columbia University, New York, NY 10032, USA; davidjiangya@gmail.com (D.J.J.); cx2209@cumc.columbia.edu (C.L.X.); 2 Edward S. Harkness Eye Institute, New York-Presbyterian Hospital, New York, NY 10032, USA; 3 Department of Pathology & Cell Biology, Stem Cell Initiative (CSCI), Institute of Human Nutrition, Vagelos College of Physicians and Surgeons, Columbia University, New York, NY 10032, USA

**Keywords:** gene therapy, gene editing, CRISPR/Cas9, Cas12a, dual AAV, triple AAV, clinical trials, retina, hereditary retinal dystrophies

## Abstract

Recently, there have been revolutions in the development of both gene medicine therapy and genome surgical treatments for inherited disorders. Much of this progress has been centered on hereditary retinal dystrophies, because the eye is an immune-privileged and anatomically ideal target. Gene therapy treatments, already demonstrated to be safe and efficacious in numerous clinical trials, are benefitting from the development of new viral vectors, such as dual and triple adeno-associated virus (AAV) vectors. CRISPR/Cas9, which revolutionized the field of gene editing, is being adapted into more precise “high fidelity” and catalytically dead variants. Newer CRISPR endonucleases, such as CjCas9 and Cas12a, are generating excitement in the field as well. Stem cell therapy has emerged as a promising alternative, allowing human embryo-derived stem cells and induced pluripotent stem cells to be edited precisely in vitro and then reintroduced into the body. This article highlights recent progress made in gene therapy and genome surgery for retinal disorders, and it provides an update on precision medicine Food and Drug Administration (FDA) treatment trials.

## 1. Introduction

With the rising mean age of the human population and rapid advances in conventional medicine, we are seeing an increase in the number of diseases that take root not from environmental factors, but from the human genome itself. To address this issue, gene therapy has risen to prominence in the field of translational research. Since the first successful application of gene therapy on a four-year-old girl with severe combined immunodeficiency (SCID) in 1990, billions of dollars have been spent on the research and development of gene therapy treatments for numerous diseases [1].

The eye has been a favourable organ for both gene medicine therapy and genome surgery because it has a multitude of unique physiological and anatomical features. The eye and retina can be accessed and observed in vivo through non-invasive methods, allowing physicians to accurately monitor disease progression and treatment efficacy [2]. Advances in imaging technology have created more portable and cost-effective fundus autofluorescence imaging cameras that are simple enough to be operated by non-ophthalmic photographers [3]. Modern day spectral domain optical coherence tomography (OCT) can capture images of the retina at the scale of microns, allowing precise visualization of the individual layers of the retina [4]. Whereas previously limited by safety concerns due to light exposure, OCT imaging can now take tens of thousands of images every second, allowing three-dimensional scans of the retina to be generated [3]. If disease progression occurs symmetrically, the untreated eye, as one of the few naturally paired organs, can also serve as an ideal control [4]. The eye is uniquely immune-privileged for a number of reasons [5]. The blood retina barrier and a lack of lymphatic vessels which prevent free travel of cells and other molecules into and out of the eye, the presence of immunosuppressive factors in the vitreous humor, and the active regulation of systemic immune responses to prevent inflammation all contribute to the eye’s ability to tolerate gene therapy [6,7].

With a prevalence of 1/3000 and caused by mutations in over 200 genes identified thus far, hereditary retinal dystrophies (HRD) are one of the most studied disease groups in gene therapy [8]. Early preclinical trials, such as the successful rescue of retinal degeneration in mouse models of retinitis pigmentosa (RP) by Bennett et al. in 1996 and the restoration of vision in canine models of RPE65 Leber congenital amaurosis (LCA) proved the efficacy of retinal gene therapy [9,10]. These successes, along with the recent, promising advent of clustered regularly interspaced short palindromic repeats (CRISPR)-driven genome surgery, have accelerated research in the field. This article will review current and developing methods of genome surgery and genetic medicine therapy, as well as recent applications in disease-specific clinical trials.

## 2. CRISPR Genome Surgery

The discovery and recent application of the CRISPR system to in vitro and in vivo gene editing has generated great excitement in the field of gene editing. The greatest advantage of CRISPR is that it is less expensive and more efficient than techniques such as zinc-finger nucleases (ZFN) or transcription activator-like effector nucleases (TALEN) [11]. CRISPR is guided by RNA sequences, which are simpler to engineer than the complex proteins upon which ZFNs and TALENs rely [12]. CRISPR is also uniquely capable of targeting more than one genetic location via multiplexed genome surgery by packaging multiple guide RNA sequences into viral vectors [13].

CRISPR-mediated gene editing can be accomplished by three strategies: non-homologous end joining (NHEJ), homology-directed repair (HDR), or microhomology-mediated end joining (MMEJ). In NHEJ, a double-stranded break (DSB) is induced at the target site, and the break is repaired by ligation of the two ends [14]. This is an error-prone process, and the resulting insertion–deletions (indels) can effectively knock out the function of the affected gene. However, frameshift mutations can also result in novel, detrimental phenotypes. In HDR, the DSB is repaired with an exogenous template sequence which is homologous to the target site [14]. Although more precise, HDR is limited to cells in S and G2 phase of cell cycle, with efficiency reduced to the point of inefficacy in post-mitotic cells. MMEJ is a recently developed form of end joining, a subset of alternative-nonhomologous end joining (A-NHEJ) [15]. It is an error-prone repair process that utilizes the alignment of microhomologous regions of the broken DNA ends before ligation.

CRISPR has been the focus of much translational research. First utilized in 2012 by Jinek et al., the CRISPR/Cas9 system of *Streptococcus pyogenes* (SpCas9) makes use of crRNA and tracrRNA combined into a chimeric “single guide” RNA (sgRNA), and it was found to create DSBs with high specificity [16]. In 2016, Latella et al. demonstrated that CRISPR/Cas9 successfully edited the human rhodopsin gene in vivo in mouse retinas [17]. In 2017, Tsai et al. showed that the “ablate and replace” approach to gene editing, in which the mutated gene is replaced with the desired gene without affecting normal function, was more effective than simple gene replacement [18]. Studied in autosomal dominant rhodopsin-associated RP mouse models, the outer nuclear layer (ONL) in mice treated with ablate and replace was 16–17% thicker than in those treated with gene replacement-only therapy. The ablate and replace technique is also mutation-independent, usable for all types of mutations in the same gene, and it is thus a faster and cheaper gene editing strategy. In addition to the treatment of diseases, CRISPR/Cas9 has also been used to create accurate mouse models for RP and LCA [19].

Despite its successes, the widely researched CRISPR/Cas9 system still has its drawbacks. SpCas9, which measures 4.2 kilobases (kb) in length, is fairly large and cannot be easily packaged into a single adeno-associated virus (AAV) vector along with its accompanying sgRNA [20]. Typically, it is necessary to employ a dual vector system, with one vector carrying the SpCas9 sequence and the other carrying the sgRNA. The efficiency and accuracy of CRISPR/Cas9 genome surgery is also of concern. In vivo testing has shown that NHEJ repair of CRISPR/Cas9-induced DSBs is only successful at a rate of 1–10%, and HDR efficiency is even lower. The SpCas9 endonuclease and sgRNA can tolerate up to five base mismatches, resulting in off-target effects and mutagenesis that occurs at frequencies comparable to on-target edits [21]. Lastly, the proper protospacer adjacent motif (PAM) sequences are within 3 bp adjacent to the cleavage site, creating strict limitations on where SpCas9 can induce DSBs.

However, much has been done to increase the efficacy and safety of CRISPR/Cas9. One potential solution is nicking enzymes (SpCas9n), which produce single-strand breaks (SSB) that are repaired with greater fidelity in eukaryotic cells than the DSBs produced by regular SpCas9 [22]. Another is the “high fidelity” Cas9 variant SpCas9-HF1, which was shown by Kleinstiver et al. to retain on-target transduction levels comparable to those achieved by SpCas9 while creating no genome-wide off-target effects [23]. To address the inefficiency of HDR, especially in non-dividing cells, Suzuki et al. demonstrated in 2016 a novel CRISPR Cas9 technique, homology-independent targeted integration (HITI) [24]. By cleaving both the DNA target site and both ends of the DNA insert, the insert can be ligated into the target site to create a precise knock-in without the HDR pathway. Even if integration occurs in the opposite orientation, it is possible to remove the insert using Cas9 until the correct orientation is achieved. This technique displayed higher knock-in efficiencies than HDR both in vitro in HEK293 cells and in vivo in RP mouse models. Furthermore, the catalytically dead Cas9 endonuclease coupled with a guide RNA, known as CRISPR interference (CRISPRi), has been shown to successfully repress target genes by silencing transcriptional activity without significant off-target effects [25]. Non-CRISPR approaches to transcriptional silencing have also proven to be effective in the retina. In 2011, Mussolino et al. demonstrated that delivery of artificial zinc-finger proteins (ZF-R6) into photoreceptors (PR) effectively suppressed the human rhodopsin (*hRHO*) gene in retinitis pigmentosa mice models [26]. In 2016, Botta et al. successfully suppressed *Rho* expression in pig retina using the zinc-finger protein ZF6-DB [27].

As mentioned previously, the relatively large size of SpCas9 makes the packaging of it and sgRNA into a single AAV vector infeasible in most cases. The smallest Cas9 orthologue, however, derived from *Campylobacter jejuni* (CjCas9), has been shown by Kim et al. to be effective in vitro and in vivo when packaged into an all-in-one single AAV vector [28]. Paired with GX22sgRNAs that hybridize with a 22 nucleotide target DNA sequence upstream of the PAM, the co-transfection of CjCas9 into HEK293 cells and NIH3T3 cells induced indels at an average frequency of 21 ± 5%. AAV9 vectors encoding CjCas9 paired with U6 promoter-derived sgRNA targeting the *Vegfa* and *Hif1a* genes, whose expression is linked with choroidal neovascularization (CNV), were administered to mouse retinas. In the retinal pigment epithelium (RPE), AAV-CjCas9: *Vegfa* induced indels at *Vegfa* and *Hif1a* sites with average frequencies of 20 ± 5% and 58 ± 12%, respectively. VEGFA protein levels in the retina, measured with enzyme-linked immunosorbent assay (ELISA), decreased after administration of AAV-CjCas9: *Vegfa* and AAV-CjCas9: *Hif1a*, and protein levels in the RPE decreased after administration of AAV-CjCas9: *Vegfa*. No off-target indels were detected in the *Vegfa* and *Hif1a* genes, indicating that the suppression of VEGFA was not an off-target effect. CjCas9 gene editing also partially prevents the development of CNV in mouse retinas subject to laser treatment. Treated with AAV-CjCas9: *Vegfa* and AAV-CjCas9: *Hif1a*, the area of CNV in the RPE was reduced by 24 ± 4% and 20 ± 4%, respectively. To test safety, AAV-CjCas9: *Vegfa* and AAV-CjCas9: *Hif1a* were also administered in CNV-free mice. ERG analysis showed that neither AAV-CjCas9: *Vegfa* nor AAV-CjCas9: *Hif1a* caused decreases in photopic response or 30 Hz flicker response. However, opsin-positive area was reduced by 30 ± 10% after administration of AAV-CjCas9: *Vegfa*, suggesting that partial knockout of *Vegfa* can cause opsin dysfunction near RPE-transduced cells. AAV-CjCas9: *Hif1a* did not cause these problems.

CRISPR Cas12a (Cpf1) is a recently reported class II/type V CRISPR endonuclease discovered in *Prevoltella* and *Francisella 1* bacteria [29]. Cas12a offers some key advantages over Cas9. At 3.7 kb, its smaller gene size enables packaging into a single AAV vector [30]. Cas12a requires only a single crRNA sequence, unlike Cas9, which is guided by both crRNA and tracrRNA. Thus, it requires a shorter gRNA sequence than the sgRNA used by Cas9. Cas9 requires tracrRNA to process crRNA, but Cas12a exhibits ribonuclease activity that enables its precursor crRNA to be processed into mature crRNA. This shorter crRNA used by Cas12a, measuring around 42 nucleotides (nt), is cheaper to design and easier to deliver than the longer sgRNA sequence used by Cas9, which measures around 100 nt [29]. This is especially useful in simplifying multiplex genome editing, and furthermore, up to four genes in mammalian cells and three genes in mouse brains can be edited simultaneously with a single crRNA array [31]. Cas12a cleavage creates a staggered cut with a 5’ overhang, instead of the blunt ends generated by Cas9 [29]. This allows for the engineering of specific sticky end sequences, which results in more precise NHEJ repair, a promising alternative to HDR. Cas12a also cleaves DNA distal from the PAM (~18 nt), preserving the target site and allowing for subsequent administration of Cas12a for a second round of cleavage. The ability for repeated cleavage enhances HDR, as demonstrated by Moreno-Mateos et al. in zebrafish [32]. In two of the four tested loci, administration of *Lachnospiraceae bacterium ND2006* Cpf1 (LbCpf1) significantly improved the efficiency of HDR (up to 4-fold) compared to SpCas9. Lastly, Cas12a recognizes a T-rich PAM sequence, unlike all other characterized gene-editing proteins, which require at least one G, and thus, Cas12a expands the targeting range of RNA-guided endonucleases. Preliminary studies show that Cas12a is a promising alternative to Cas9. Using a two plasmid-based editing approach, LbCpf1 and *Francisella novicida U112* Cpf1 (FnCpf1) displayed editing efficiencies comparable with Cas9 in yeast cells [33]. Koo et al. found that Cas12a induced indels at high frequencies in *Vegfa* and *Hif1a* genes in mouse models, which led to a long-term reduction of areas of laser-induced CNV without causing cone-dysfunction [30]. At six weeks post intravitreal injection, AAV2/9 expressing *Vegfa*-specific LbCpf1 (AAV-Cpf1-Vegfa) induced indels at an average frequency of 57.2 ± 4.1% in the retina and 6.5 ± 2% in the RPE, and AAV2/9 expressing *Hif1a*-specific LbCpf1 induced indels at a frequency of 59.2 ± 4.9% in the retina and 7.2 ± 5.3% in the RPE.

Since the early 2000s, researchers have been developing ways to harness CRISPR and create base-editing enzymes, capitalizing on Cas9’s RNA-guided site specificity while blocking it from creating double-stranded breaks. Fusing a catalytically dead Cas9 (dCas9) to rAPOBEC1, a cytidine deaminase enzyme, creates a base-editing enzyme (BE1) that can convert cytosine to uracil in a precise and site-specific manner [34,35]. Using BE1 converts C:G to U:G, and in order to block DNA repair mechanisms from changing U:G back to C:G, adding a uracil DNA glycosylase inhibitor (UGI) to BE1 allows manipulation of the DNA repair system to generate T:A base pairs from U:G at a high yield [36,37]. This rAPOBEC1-XTEN-dCas9-UGI complex is called “BE2”. Modifying Cas9 into a nicking enzyme by restoring the position 840 His in the catalytic HNH domain of BE2 generates APOBEC-XTEN-dCas9(A840H)-UGI, or BE3 [38,39]. BE3 improves base editing efficiency and yields more U:A product. All three aforementioned base editors mediate conversion of C to T. In order to mediate base conversion of A to G and to advance the scope of genetic diseases that can be researched and potentially treated with base editors, Gaudelli et al. developed adenine base editors (ABEs) [40]. Since there are no naturally occurring enzymes which deaminate adenine, they utilized directed evolution to create a tRNA adenosine deaminase which can mediate conversion of A to G when fused to dCas9.

## 3. Gene Therapy and Stem Cell Therapy

Unlike genome surgery, gene augmentation therapy does not directly modify the existing genome. Instead, it supplements the endogenous genome with a wild-type copy of the defective gene. It was first demonstrated in 1996 to successfully rescue photoreceptor cells in a retinal degeneration mouse model, and a few years later to rescue retinal function in Leber congenital amaurosis (LCA) canine models [9,10]. Translated to clinical trials, gene therapy aimed at retinal diseases has been repeatedly shown to be safe and efficacious in humans [8]. However, gene augmentation therapy treatments are limited to autosomal recessive diseases.

Another option is gene therapy via stem cell transplantation, in which cells are edited in vitro using genome surgical techniques such as CRISPR/Cas9 (far more precisely than in vivo) and then introduced into the body. The use of embryonic stem cells (ESC) has been proven to be safe and efficacious in both preclinical and clinical trials, with research for retinal disorders starting in 2010 [40]. ESC-derived RPE cells transplanted into MERTK retinal degeneration rat models improved PR function and increased visual function [41,42,43]. Lu et al. found that human ESC (hESC)-derived RPE cells survived for 200 days in rat models and improved computer-assessed visual function [44]. Although the use of hESCs has been shown to be safe and efficacious in humans, the potential for immunological responses and ethical controversies have raised concerns over their usage [45]. A promising alternative is fibroblast-derived induced pluripotent stem cells (iPSCs), which would both significantly decrease the risk of immunological rejection and also circumvent the ethical criticism of hESCs. Multiple studies have found that administration of iPSCs in mouse models safely produces improvements in retinal function [46,47,48]. In 2016, Bassuk et al. edited iPSCs from an X-linked retinitis pigmentosa (XLRP) patient with high precision in vitro using CRISPR/Cas9 [49]. Of the RPGR genes, 13% were successfully corrected. However, the abnormal genetic composition of iPSCs may elicit T-cell immune responses [50]. Additionally, tumor formation may result from incompletely differentiated iPSCs [40], and a clinical trial in Japan was discontinued in part due to genetic abnormalities detected in transplanted iPSCs [51]. A third option is the use of mesenchymal stem cells (MSCs), multipotent cells that are found in various adult tissues and have been shown to be able to differentiate into a variety of retinal cell types, including RPE, PR, bipolar, amacrine, and Müller glial cells. Their strong immunosuppressive effects mean that both autologous and allogenic transplantations are possible, and, unlike iPSCs, they do not cause tumor formation [52]. Phase I clinical trials have shown that intravitreal injection of bone marrow-derived MSCs are well tolerated in patients [53,54], but sight-threatening vitreoretinal complications, such as retinal detachment, have been noted in case reports [55,56].

## 4. Vector Choice

Currently, viral vectors are predominantly used to deliver genetic material into cells for gene editing and therapy. Among them, the adeno-associated virus (AAV) vector is used most commonly and has been demonstrated to be safe in an overwhelming majority of clinical trials [57]. AAVs are especially advantageous because they are not pathogenic and they lack lipids and other immunological response-inducing compounds. AAVs are often paired with expression cassettes that increase target specificity and transgene expression levels for specific diseases. In particular, cell-specific promoter sequences greatly increase target specificity, and they are critical for preventing off-target mutagenesis. However, AAV vectors are frequently limited by their size, because they can only package genes up to 4.5 kb long [58]. This is insufficient for certain diseases, such as Usher Syndrome Type 1B (USH1B) caused by mutations in the *MYO7A* gene (of which the cDNA is 7 kb long), and Stargardt disease caused by mutations in the *ABCA4* gene (of which the cDNA is 7.3 kb long).

Dual AAV vectors, in which different parts of the gene package are stored in separate AAV vectors, have a total carrying capacity of up to 8.9 kb, and they are often used to address the size limitation presented by a single AAV vector [59]. This technique utilizes the inherent ability of AAV genomes to concatemerize, and once inside the cell, the reconstitution of the full length gene is achieved upon co-infection of the same cell [60]. Dual AAV vectors have been shown to safely and effectively transduce photoreceptors and improve retinal functions in Stargardt 1 (STGD1) and Usher1b (USH1B) mouse models without creating the potentially dangerous truncated proteins that typically manifest with oversized single AAV vectors [60,61,62]. However, the carrying capacity of dual AAV vectors is still insufficient for diseases such as Usher 1D, which is caused by mutations in the 10.1 kb *CDH23* gene. For such diseases, triple AAV vectors, which can package DNA sizes up to 14 kb, are a potential solution. In 2017, Maddalena et al. studied the transduction of PRs through administration of triple AAV2/8 vectors carrying the ED reporter protein [63]. Although transduction of mouse PRs only obtained 2 ± 1% of that observed with single AAV vectors, in pig retinas, protein expression in the PRs was 39 ± 17%. The poor transduction of mouse PRs compared to pig PRs was also observed when studying the efficiency of dual AAV vectors. Other viable viral vectors include the lentivirus (LV) with a carrying capacity of ~9 kb, the adenovirus (Ad), and the herpesvirus (HV) ~150 kb [64]. Preclinical trials have shown that LV vectors successfully delivered corrected copies of the *ABCA4* gene and *MYO7A* gene into Stargardt and USH1B mouse models [65,66]. However, these high capacity vectors, including LV, do not transduce PRs nearly as effectively as AAV2/8, and they also have higher immunogenicity [64].

## 5. Delivery Method

Gene therapy agents can be delivered into the eye either via subretinal injection or intravitreal injection. In intravitreal injections, the agent is delivered into the vitreous cavity, and it primarily infects the ganglion cell layer. While easier to perform and less risky, this method is more likely to cause immunological responses, because of a greater systemic spread of the agent [67,68]. Subretinal injections deliver the agent in a more precise and localized manner and they are effective at targeting RPE and PR cells. However, they are more invasive than intravitreal injections, and the injection bleb must be carefully controlled in order to prevent the development of macular holes and retinal detachment [68]. Additionally, the surgeries require highly advanced training, and the integrity of the eye must be sufficiently stable before subretinal injections can be performed. The recent development and spread of surgical assistance robotic systems, such as the Intraocular Robotic Interventional Surgical System (IRISS), developed by the Jules Stein Eye Institute and the University of California Los Angeles, and the Robotic Retinal Dissection Device Trial (R2D2), developed by the University of Oxford, hold the potential to greatly increase the precision, safety, and simplicity of such procedures [69] (ClinicalTrials.gov #NCT03052881).

## 6. Recent Developments in Disease-Specific Clinical Trials

For a summary of current clinical trials for diseases discussed in the following section, see Table A1 in Appendix A.

### 6.1. Leber Congenital Amaurosis 2 (LCA2)

Leber congenital amaurosis 2 is an early onset retinal dystrophy that is characterized by poor vision, nystagmus, sluggish pupillary responses, and photophobia. It usually results in severe vision impairment in the first year of life, and it occurs in 2–3 per 100,000 births [70]. LCA2 is caused by mutations in the *REP65* gene and affects patients at a rate of 3–16%. In phase I and II clinical trials, unilateral administration of rAAV2 vectors carrying corrected copies of the *RPE65* gene have been demonstrated to improve visual function for up to three years [71,72,73]. In 2012, Bennett et al. demonstrated that contralateral subretinal re-injection of rAAV2-hRPE65v2 safely resulted in improved visual function with no detectable immunological responses [74]. Encouraged by these findings, Spark Therapeutics conducted a phase III clinical trial testing bilateral administration of voretigene neparvovec (rAAV2-hRPE65v2), and functional vision improved in the intervention group [75]. In 2017, Luxturna (voretigene neparvovec-rzyl), a single-use gene therapy drug, became the first Food and Drug Administration (FDA)-approved gene therapy for a genetic disorder [76]. Spark Therapeutics continues to conduct clinical trials to determine the long-term effects of voretigene neparvovec-rzyl (ClinicalTrials.gov #NCT03602820, #NCT01208389, #NCT00516477).

### 6.2. Leber Congenital Amaurosis 10 (LCA10)

Among the subtypes of LCA, LCA10 is the most common, affecting LCA patients at a rate of 20–30% [77,78]. It is caused by genetic mutations in *CEP290*, and the most frequent LCA10 mutation is IVS26 c. 2991+1655 A>G, one that creates a cryptic splice site. In 2015, Maeder et al. used SaCas9 (a CRISPR system derived from *Staphylococcus aureus*), to deliver two gRNAs into human fibroblasts from LCA patients with homozygous IVS26 mutations [79]. The gRNAs guided the excision of the mutation-containing DNA region with two double-stranded breaks, and the CRISPR-corrected cells demonstrated an increase in wild-type *CEP290* expression.

In 2017, Editas Medicine reported successful in vivo editing of *CEP290* in transgenic mice containing the human ISV26 mutation [77,80]. Their CRISPR machinery, called “EDIT-101,” contains two gRNAs and SaCas9 packaged into AAV5 vectors, and it is delivered into mice retina via subretinal injection. EDIT-101 demonstrated efficient transduction of photoreceptors, rapid onset, and stable editing that lasted throughout six months of observation post-injection.

In 2018, Dulla et al. demonstrated that their QR-110 oligonucleotide, designed to correct the ISV26 splicing defect, successfully restored wild-type *CEP290* mRNA and protein expression levels in LCA10 fibroblasts and retinal organoid models [81]. Intravitreal delivery of QR-110 was shown to penetrate all cellular layers of the retina in animal models, and it was well tolerated for up to 28 days in non-human primates. ProQR Therapeutics is currently conducting a phase I/II trial to study the safety and tolerability of intravitreally administered QR-110, with a primary completion date of December 2019 (ClinicalTrials.gov #NCT03140969). Initial findings show that QR-110 was well tolerated with no serious adverse effects and resulted in visual improvement in approximately 60% of subjects [82].

### 6.3. X-Linked Forms of Retinitis Pigmentosa (XLRP)

Retinitis pigmentosa (RP) causes loss of photoreceptor function and is characterized by night blindness and progressive loss of visual fields [83]. It occurs with a prevalence of approximately 1 in 3500 births. XLRP accounts for 10–20% of RP, and 70% of XLRP is caused by mutations in the *RPGR* gene. AAV2/5 deliverance of full-length human *RPGR^ex1-ORF15^* prevented onset of photoreceptor degeneration in XLPRA1 canine models and rescued photoreceptor and postreceptoral ERG function in XLPRA2 canine models [84]. A clinical trial conducted by MeiraGTx UK II Ltd. (London, UK), is testing the safety and efficacy of administration of AA2/5-hRKp.RPGR in patients with diagnosed XLRP (ClinicalTrials.gov #NCT03252847). It has a primary completion date by November 2020 and has recently been fast-tracked by the FDA [85]. Nightstar Therapeutics and Applied Genetic Technologies Corporation are both also conducting phase I/II trials studying administration of an AAV carrying the corrected RPGR gene (ClinicalTrials.gov #NCT03314207, #NCT03116113).

### 6.4. Age-Related Macular Degeneration (AMD)

Characterized by the loss of RPE and PR cells, AMD is the third-leading cause of blindness in the world and occurs in 13% of the population over the age of 85 [86]. Gene therapy for wet AMD (wAMD), in which neovascularization occurs between the RPE and the retina, focuses on long-term inhibition of vascular endothelial growth factor (VEGF). In 2013, a phase I/II trial found that subretinal injections of AAV vectors carrying the VEGF inhibitor *sFLT-1* (rAAV.SFLT-1) were well tolerated in elderly wAMD patients and resulted in no drug-related adverse effects [87]. Additionally, the majority of patients displayed improved visual function, indicating that the treatment is potentially efficacious. Oxford BioMedica found in a phase I trial that administration of RetinoStat—a lentiviral Equine Infectious Anemia Virus (EIAIV) vector expressing the endostatin and angiostatin proteins—was safe and well tolerated, and it resulted in expression that lasted more than four years in 2 of the 4 patients [88]. Regenxbio Inc. is currently conducting a phase 1 trial studying subretinal administration of RGX-314, an AAV8 vector carrying a gene encoding for a soluble anti-VEGF protein, with an estimated completion date of September 2020 (ClinicalTrials.gov #NCT03066258).

Stem cell therapy is another promising strategy in treating AMD. Results for a phase I trial published in 2018 showed that 2 patients with severe wAMD were administered hESC-derived RPE transplants and had improved visual function for 12 months [89]. In 2017, Mandai et al. demonstrated that transplantation of iPSC-derived RPE cells in a patient with wAMD resulted in no immunological rejection even without the administration of immunosuppressants [90]. Furthermore, the patient experienced stable vision one year after transplantation. Stem cell therapy is also potentially efficacious in treating atrophic (dry) AMD as well, which currently has no treatment but accounts for 80–90% of AMD cases [91]. Results from a phase I/II trial testing the safety and tolerability of hESC-derived RPE patches transplanted subretinally in dry AMD patients were published in 2015. Patients were observed for a median of 22 months and up to 37 months. The transplants were well tolerated, with no teratoma formations, immune reactions, or differentiation of cells into unwanted types. At six months, visual acuity increased by at least 15 letters in 4 eyes, 11–14 letters in 2, and remained stable in 3. At 12 months, of the 7 patients followed up, visual acuity increased by at least 15 letters in 3 eyes, 13 letters in 1, and remained stable in 3. In 2018, Kashani et al. reported successful transplantation of CPCB-RPE1, a bioengineered monolayer of hESC-derived RPE cells, in advanced dry AMD patients [92]. Of the 4 subjects out of 5 who successfully received the implant, all of them maintained vision, and one subject’s eye improved by 17 letters.

### 6.5. Choroideremia

Choroideremia is an X-linked disorder that causes loss of RPE, choroid, and PR cells [93]. It begins with early onset night blindness, advances with progressive constriction of vision, and results in complete blindness in the late stages of life [94]. It has a prevalence of 1 in 50,000 people of European descent, and it is caused by mutations in the *CHM* gene. Subretinal administration of AAV2 vectors encoding the REP1 protein (AAV2.*REP1*) was demonstrated by MacLaren et al. to increase retinal sensitivity in 5 out of 6 patients and substantially improve visual acuity and rod and cone function in the 2 patients with the most advanced choroideremia [95]. Increased sensitivity of the treated eye correlated with the vector dose per area of live retinal cells. A follow-up study demonstrated that by 3.5 years, the treated eyes experienced improvements, whereas the control eyes continued to deteriorate. Nightstar Therapeutics is conducting a phase III clinical trial administering AAV2.REP1 to 140 choroideremia patients, with a completion date of March 2020 (ClinicalTrials.gov #NCT03496012). Spark Therapeutics is conducting a phase I/II studying the safety and tolerability of subretinal administration of AAV2-hCHM, with an enrollment of 15 patients and a completion date of January 2019 (ClinicalTrials.gov #NCT02341807).

### 6.6. Usher Syndrome

Usher syndrome 1 (USH1) is characterized by congenital, bilateral hearing loss and early-onset retinitis pigmentosa [96]. It has a prevalence of 3.0–6.2 per 100,000 and is caused by mutations in six identified genes [4]. Among them, USH1B, caused by mutations in the *MYO7A* gene, is being studied in gene therapy trials. In a phase I/II trial sponsored by Sanofi, UshStat, an EIAV lentiviral vector carrying the wild-type *MYO7A* gene, is being administered subretinally to 18 patients with either Usher syndrome or retinitis pigmentosa (ClinicalTrials.gov #NCT01505062). The conclusion of the trial is expected in January 2021.

### 6.7. Stargardt Disease

Stargardt disease is characterized by early onset bilateral central vision loss, macular atrophy, and progressive loss of outer retinal function and structure. With a prevalence of 1:10,000, it is the most common hereditary macular dystrophy and is caused by mutations in the *ABCA4* gene [97]. Although too large to be packaged into a single AAV vector, the corrected human *ABCA4* gene has been delivered via EIAV lentivirus vectors in mouse models in preclinical trials, leading to the successful transduction of genetic information in 5–20% of PR cells and the reduction of lipofuscin pigment A2E accumulation [66]. Preliminary results of a Sanofi-sponsored phase I/II clinical trial administering subretinal doses of a lentivirus vector carrying a corrected copy of the *ABCA4* gene (SAR422459) in Stargardt patients are positive, with patients experiencing no adverse effects (ClinicalTrials.gov # NCT01367444). Another phase I/II trial was conducted to study the tolerability and safety of subretinal transplantation of hESC-derived RPE cells (ClinicalTrials.gov #NCT01345006). Of the Stargardt patients, 9 were observed for up to 37 months, and no adverse effects, such as teratoma formations, immunological responses, or unwanted differentiation of cells, occurred. Additionally, 3 of 8 observed patients had improved visual acuity after six months, and 3 out of 7 observed patients had improved visual acuity after twelve months.

### 6.8. Leber Hereditary Optic Neuropathy (LHON)

LHON is characterized by loss of the ganglion cell layer, resulting in subacute vision failure in young adult life [98]. It has an estimated prevalence of between 1 in 31,000 and 1 in 50,000 and is most commonly caused by mutations in the *ND1*, *ND4*, or *ND6* genes, which code for a key enzyme affecting mitochondrial function [99]. In a phase III trial currently sponsored by GenSight Biologics, GS010, an AAV2 vector encoding the human wild-type *ND4* gene, is being intravitreally administered to G11778A ND4 LHON patients (ClinicalTrials.gov #NCT03293524). Preliminary results demonstrate that no severe adverse effects occurred to 5 legally blind patients [100]. As measured by the Early Treatment Diabetic Retinopathy Study (ETDRS), 2 patients experienced an increase in visual acuity.

## 7. Conclusions

The rapid development of gene medicine therapy and genome surgery holds the potential to revolutionize the field of medicine, and its advances in the past decades have been remarkable. The eye, already at the forefront of gene medicine therapy, has become more accessible than ever with the development of advanced imaging and surgical technology. Conventional gene augmentation therapy continues to increase in both safety and efficacy as researchers have developed improved methods of using viral vectors. The emergence of dual and triple AAV vectors have addressed the size limitation of the gold standard rAAV vector system, and the successes of a lentiviral vector in treating USH1B have greatly increased the toolkit of vectors available to scientists.

To treat autosomal dominant diseases, genome surgery using the CRISPR/Cas9 system has generated promising results. Yet, despite its successes, safety remains a concern due to potential off-target mutagenesis, especially as CRISPR makes its way into human clinical trials. The FDA has only recently lifted a hold placed on the first CRISPR clinical trial in the United States (US) [101], which aims to study the safety and efficacy of CRISPR-Cas9 modified CD34+ human hematopoietic stem and progenitor cells in patients with transfusion-dependent β-thalassemia (ClinicalTrials.gov #NCT03655678). A European trial testing the same treatment in conjunction with the US trial is on track to begin in late 2018. In China, CRISPR clinical trials have already been running and numerous patients have been treated.

As discussed in this review article, the development of newer non-cutting CRISPR technology seeks to increase the precision and safety of the system. “High fidelity” Cas9 strands and CRISPR interference have been shown to increase precision without compromising efficacy, and CjCas9 and Cpf1 CRISPR endonucleases have shown great promise. Stem cell therapy has also emerged as an alternative, and the development of iPSC treatments addresses both the controversy and immunogenicity concerns of traditionally used hESCs. Such discoveries and developments will continue to generate excitement in the field of genetics research and will continue to push modern medicine towards a paradigm shift that will see the application of gene therapy and surgery in common treatments.

## Appendix A

**Table A1:** Current Gene Medicine Therapy Clinical Trials for Inherited Retinal Disorders.

Disease	Treatment	Phase	End Date	NCT ID and Sponsor
Leber congenital amaurosis 2 (LCA2)	Subretinal Administration of AAV2-hRPE65v2	III	2029	NCT00999609 Spark Therapeutics
		I	2024	NCT00516477 Spark Therapeutics
		I/II	2026	NCT01208389 Spark Therapeutics
		I/III	2030	NCT03602820 Spark Therapeutics
	Subretinal Administration of AAV2/5 OPTIRPE65	I	2018	NCT02781480 MeiraGTx UK II Ltd.
	Subretinal Administration of AAV2/5 OPTIRPE65	I/II	2023	NCT02946879 MeiraGTx UK II Ltd.
	Subretinal Administration of rAAV2-CBSB-hRPE65	I	2026	NCT00481546 University of Pennsylvania
LCA10	Intravitreal Administration of QR-110	I/II	2018	ProQR Therapeutics
Retinitis Pigmentosa (RP)	Intravitreal Administration of RST-001	I/II	2033	NCT02556736 Allergan
	Subretinal Administration of rAAV2-VMD2-hMERTK	I	2023	NCT01482195 Fowzan Alkuraya
	Subretinal Administration of AAV2/5-hPDE6B	I/II	2022	NCT03328130 Horama S.A.
X-Linked Retinitis Pigmentosa (XLRP)	Subretinal Administration of rAAV2tYF-GRK1-RPGR	I/II	2024	NCT03316560 Applied Genetic Technologies Corp
	Subretinal Administration of AAV2/50hRKp.RPGR	I/II	2020	NCT03252847 MeiraGTx UK II Ltd.
	Subretinal Administration of AAV-RPGR	I/II	2019	NCT03116113 Nightstar Therapeutics
Neovascular Age-Related Macular Degeneration (AMD)	Subretinal Administration of RGX-314	I	2020	NCT03066258 Regenxbio Inc.
	Intravitreal Administration of AAV2-sFLT01	I	2018	NCT01024998 Sanofi (Genzyme)
Atrophic AMD	RPE Transplantation	I/II	2020	NCT02755428 Chinese Academy of Sciences
Choroideremia	Subretinal Administration of AAV2-REP1	II	2021	NCT02553135 Bryon Lam
	Subretinal Administration of AAV2/REP1	II	2021	NCT02407678 University of Oxford
	Subretinal Administration of AAV2-hCHM	II	2019	NCT02671539 Spark Therapeutics
	Subretinal Administration of rAAV2.REP1	II	2018	NCT02671539 STZ Eyetrial
	Subretinal Administration of AAV2-REP1	III	2020	NCT03496012 Nightstar Therapeutics
Usher Syndrome	Subretinal Administration of UshStat (EIAV-CMV-MYO7A)	I/II	2021	NCT01505062 Sanofi
		I/II	2036	NCT02065011 Sanofi
Stargardt Disease	Subretinal Administration of SAR422459	I/II	2019	NCT01367444 Sanofi
			2034	NCT01736592 Sanofi
Leber Hereditary Optic Neuropathy (LHON)	Intravitreal Administration of GS010 (rAAV2/2-ND4) vs. Sham Intravitreal Administration	III	2019	NCT02652767 GenSight Biologics
			2019	NCT02652780 GenSight Biologics
			2021	NCT03293524 GenSight biologics
	Intravitreal Administration of scAAV2-P1ND4v2	I	2019	NCT02161380 John Guy, University of Miami

Notes: Adeno associated virus (AAV); Recombinant adeno associated virus (rAAV).
